# Digital Twins for Healthcare Using Wearables

**DOI:** 10.3390/bioengineering11060606

**Published:** 2024-06-13

**Authors:** Zachary Johnson, Manob Jyoti Saikia

**Affiliations:** Department of Electrical Engineering, University of North Florida, Jacksonville, FL 32224, USA

**Keywords:** digital twins, wearables, AI, sensors, healthcare

## Abstract

Digital twins are a relatively new form of digital modeling that has been gaining popularity in recent years. This is in large part due to their ability to update in real time to their physical counterparts and connect across multiple devices. As a result, much interest has been directed towards using digital twins in the healthcare industry. Recent advancements in smart wearable technologies have allowed for the utilization of human digital twins in healthcare. Human digital twins can be generated using biometric data from the patient gathered from wearables. These data can then be used to enhance patient care through a variety of means, such as simulated clinical trials, disease prediction, and monitoring treatment progression remotely. This revolutionary method of patient care is still in its infancy, and as such, there is limited research on using wearables to generate human digital twins for healthcare applications. This paper reviews the literature pertaining to human digital twins, including methods, applications, and challenges. The paper also presents a conceptual method for creating human body digital twins using wearable sensors.

## 1. Introduction

The increasing trend towards digitalization allows for great technological advancements in every industry. This has been furthered by the Internet of Things (IoT); a concept that involves the interconnection of physical systems using smart sensors that can exchange information with each other [1]. This results in an ecosystem of smart devices that exists as a collective entity [2]. With the shift towards digitalization comes a heightened interest in digitally modeling physical objects by leveraging the capabilities of smart devices [3]. Digital twins are an extension of digital modeling that have produced great impacts in recent times across multiple industries [4]. Digital twins have allowed for a greater connection between the physical and digital environments by allowing both to affect one another. The digital twin is created and updated by its physical counterpart, which can then in turn be used to change and improve the physical subject of interest [5,6]. This has allowed for great improvements in product quality, effectiveness, and longevity in many industries [7,8,9,10].

**Figure 1:**
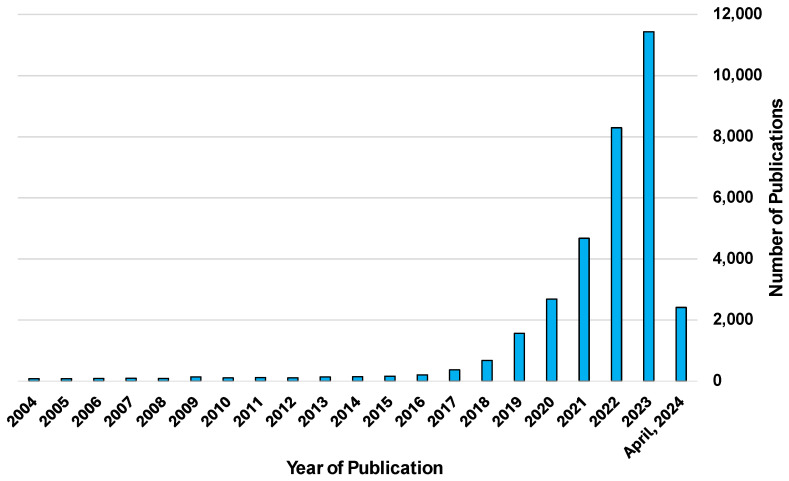
Chart detailing a list of articles with the key term “Digital Twin” by year of publication. Source: Engineering Village, 2024 Reed Elsevier.

**Figure 2:**
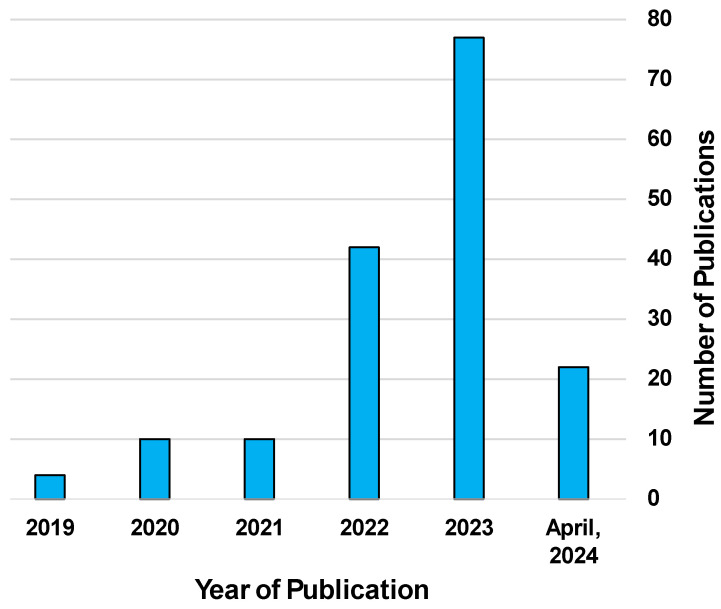
Chart detailing a list of articles with the key term “Human Digital Twin” by year of publication. Source: Engineering Village, 2024 Reed Elsevier.

Digital twins are a relatively new method of creating advanced models that take advantage of the capabilities provided by the IoT. Digital twins are a digital model created based on a physical entity that can be used for simulations to predict the outcomes of its real-life counterpart [11]. These digital models are created using data gathered from sensors and manually input to accurately capture the characteristics and properties of the desired subject [12]. What sets digital twin technology apart from other models is their ability to update in real time according to sensor data from their physical counterparts [13,14]. This allows the model to change and evolve along with its physical counterpart. This is an especially useful quality for studying dynamic systems, such as the degradation of machine components [15,16] or the treatment progression of medical patients [17]. This model can then be used in tandem with AI and machine learning algorithms to perform simulations with a great deal of precision that could be too costly or dangerous to perform on the physical model [18]. Figure 1 and Figure 2 depict the number of publications featuring the search terms “Digital Twin” and “Human Digital Twin”, respectively. Of particular interest is the noticeable increase in publications beginning in the early 2020s.

Examples of applications in healthcare include running clinical trials on digital models of patients to determine the best course of action for combating an ailment or predicting the progression of cancer for planning treatment strategies [19]. This, of course, is not limited to healthcare, as digital twin technology has affected numerous other fields such as aerospace, robotics, wireless communications, etc. [20,21]. Digital twins are also compatible with cloud technology, allowing for flexibility in monitoring and updating the model. An example of this application has been monitoring the status of a virtual patient by a clinician during a patient’s treatment plan to track the progress of symptoms [22]. These data are valuable as they allow a doctor to prescribe new medications or update treatment plans to meet a patient’s specific needs, without the patient needing to schedule a physical appointment. Notably, digital twins have enhanced the capabilities of point-of-care testing in numerous ways. Tests performed remotely can now be monitored by healthcare professionals through analytics provided by the patient’s digital twin. This has the potential to drastically expedite treatment progression as the patient no longer must schedule physical appointments to conduct lab work and patients can receive results from their tests immediately. Healthcare professionals can use the test results from the patient to recommend adjustments to their treatment plans or potentially detect problems before complications arise. An example of digital twins aiding in point-of-care testing is glucose monitoring for patients with type-1 diabetes. The digital twin generated from the patient based on wearable sensor data can monitor glucose levels to notify the patient when an insulin injection is necessary. At the same time, the patient’s healthcare team can monitor the results of the patient and recommend new treatment strategies such as new medications [23]. This, in turn, holds the potential to revolutionize the healthcare industry by allowing for personalized treatment regardless of the patient [24,25].

The healthcare field has benefited greatly from advancements in digital twin technology [26,27]. Such advancements include creating digital twins of individual organs which can be used to monitor their condition under medications. These same digital twins of organs have also been used as a model to replace damaged organs through 3D printing [28]. Typically, digital twins in healthcare are limited to this specific scope, such as an organ, limb, or section of the body [29,30]. Other applications involve modeling a specific function of the human body, such as the respiratory system [31]. Digital twins created for patients are entirely personalized to suit their needs, meaning that any treatments prescribed to the patient will be less generalized [32,33]. Examples of this include digital twins that can participate in clinical trials that factor in allergies, hereditary conditions, and additional medications the patient may also be currently taking [34,35,36]. Utilizing a patient’s digital twin can allow healthcare professionals to perform simulated clinical trials to determine the most effective treatment strategy for the patient. This takes advantage of the digital twin’s capability to represent the actual patient’s characteristics and the integration of predictive analysis in DTs. By simulating every possible treatment strategy and observing the outcomes, clinicians could determine which one has the highest rate of success without directly intervening with the actual patient. This is a much safer procedure for the patient, as there are fewer risks involved that could arise due to adverse effects of medications. Additionally, this is a much faster and more cost-efficient process than that of the “trial and error” approach. Another use of digital twin applications in healthcare involves real-time disease progression tracking. Since the digital twin is constantly being updated by sensor data from the patient, any emergency that may arise in their condition can be addressed in a timely manner that could result in life-saving measures or simply better patient outcomes [37]. Digital twins have been used as a method of tracking cancer progression to determine viable treatment options that can intervene in the early stages of disease progression. They have also been utilized in conjunction with assistive devices to help patients during stroke rehabilitation experiencing limited motor control. Digital twins of humans are virtual replicas of their human counterparts [38]. What a human digital twin exactly entails varies depending on the application. However, there has been little investigation into the creation of a digital twin representing the entire human body. This has been largely attributed to limitations in computational power as the creation of a digital twin is a rigorous endeavor that requires a great deal of processing power [39,40]. In addition, methods of acquiring the data of an entire body using smart sensors are limited [41,42]. Common methods of creating digital models of human bodies typically involve the use of an MRI or cameras [43], which would not be sufficient for creating a digital twin that needs to update in real time with the physical counterpart.

**Figure 3:**
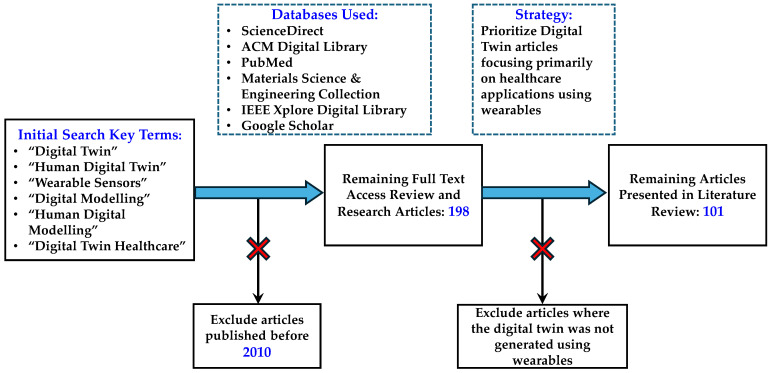
Schematic representation of the presented review process focusing on the literature pertaining to digital twins using wearables for healthcare applications.

Due to the nature of digital twins updating in real time with their physical counterparts of interest, a constant source of data is expected. As such, the quality of the digital twin is heavily reliant on the sensor used. In healthcare settings, wearable sensors provide numerous options for monitoring patients due to their versatility in what parameters they can measure and their ease of use [44,45]. Their applications range from measuring heart rate in smart watches as well as muscle activity. Smart wearable sensors also have the capability to upload the data to where they can be accessed across multiple devices and provide useful information on the patient’s general wellness. Additionally, great advancements have been made in the ease of wearing of the sensors. New materials have been incorporated into the structure of the smart wearables to make them more compact and comfortable to wear. This includes the incorporation of polymers and carbon nanomaterials as the primary component in the structure of the wearables, making them flexible and providing more efficient data acquisition [46]. As a result, they hold great potential in generating digital twins of patients by constantly providing biometric data. These data can then be constructed to make a digital twin capable of monitoring processes within the human body relevant to healthcare applications [26]. Figure 3 presents the review methodology used for selecting relevant literature pertaining to digital twins created using wearables for healthcare applications.

### Purpose

**Figure 4:**
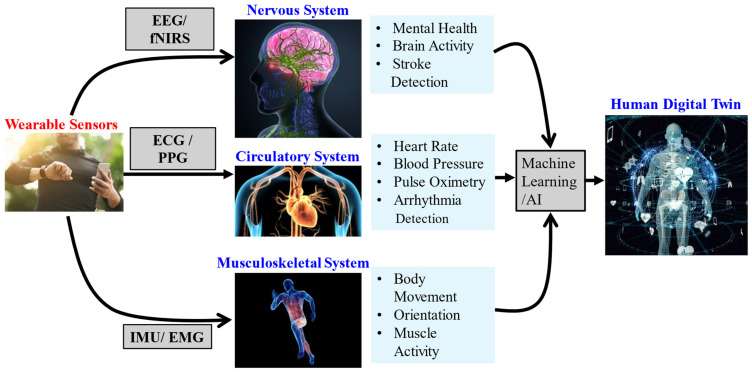
Diagram representing the proposed human digital twin generation method. This human digital twin is comprised of data gathered from the nervous, circulatory, and musculoskeletal systems.

This review paper examines different consumer-grade wearable sensors to propose a method for creating a human digital twin for healthcare. The human body is an incredibly complex collection of dynamic systems, so perfectly modeling an entire human body is not feasible with current sensors and computational capabilities. While there exists plenty of literature about digital twins of specific parts of the human body, such as organs, the focus of this review is on creating a more holistic human digital twin. Such a digital twin would include the musculoskeletal, circulatory, and nervous systems. These were chosen as each can be measured effectively using wearable sensors individually and the combination of the three can provide insight into human health. Figure 4 depicts the general acquisition process for gathering the necessary data from the systems of interest in creating a human body digital twin. These data will provide a running record of characteristics and findings which artificial intelligence can review for patterns, thus making recommendations for treatment or continued monitoring. Towards this, this paper discusses different wearable sensors that are used in the creation of human digital twins, or human digital modeling in general, and various methods for creating digital twins. 

## 2. Materials and Methods

The methodology used for this review was focused on finding relevant research in ascending degrees of specificity towards the topic of human digital twins. The goal was to find information and resources that pertained specifically to the creation of human digital twins but also more general information about digital twins and wearable sensors to provide a greater context for the review presented. As human digital twins are an interdisciplinary topic of interest, publications were chosen from different fields such as healthcare, engineering, and computer science to offer different perspectives on all the fields associated with the creation and utilization of human digital twins. This is also true for different databases to expand the scope of resources as much as possible. In addition, due to digital twins being a rapidly growing field of interest, greater priority was given to publications released within the last decade. 

Examining the literature for this review began by searching for articles using the key term “Digital Twin” to find general information regarding digital twins across various industries. This provided context behind how digital twins were being created and used in a wide variety of settings and provided insight into their similarities. This was then followed by searching for articles with “Digital Twin” and “Healthcare” to narrow the focus towards digital twins used in healthcare settings. This was important as the literature focused on human digital twins is scarce due to the infancy of digital twins as a field of interest, as well as the specificity of having humans as the subject of digital twin creation. Afterwards, more literature was found by searching with the term “Human Digital Twin”. Among the articles found on human digital twins, a substantial portion were focused on the generation of human digital twins using artificial intelligence (AI) generative data, while those using wearable sensors to generate the digital twin were uncommon. 

Our primary focus is on the creation of human digital twins using wearable sensors, and hence it was important to find the literature focused on the digital modeling of humans using wearable sensors. As a result, this led to examining articles about digital modeling in general of human bodies using wearable sensors in addition to ones focused on digital twins. While not specific to creating digital twins in particular, the process of acquiring data from wearable sensors to be used for digital modeling is still relevant to this review. Table 1 provides a list of databases and key terms used for the literature review, as well as years of publication for the relevant literature.

The process of generating a human digital twin can essentially be broken down into three steps: data acquisition, data processing, and model generation. To begin, sufficient data must be collected from the individual human on which the digital twin is based. This step begins with a smart sensor capable of acquiring the data of interest. The sensor gathers the necessary data and uploads them to a server accessible by multiple other devices where it will be processed using its on-board microcontroller unit. During the data processing step, the acquired data undergo intensive processing to extract meaningful information. This is where machine learning and AI algorithms will be utilized to detect patterns that may be of interest. Some examples of this could include discerning between normal and abnormal heart rhythms or identifying the movement patterns of people. Data encryption is also utilized during this step to protect the integrity and security of stored information. Finally, the processed data can be used to generate a model in the form of a digital twin. The model is iterative and will constantly update based on new data being received from its physical counterpart. 

## 3. Healthcare Digital Twin

### 3.1. Sensing and Data

Digital twins are created based on parameters from their physical counterparts. As such, having proper sensors is critical for generating an accurate model. Sensors are the first step in creating a digital twin through data acquisition to be used for the model. In the context of creating a human digital twin, there is a great benefit to using wearable sensors compared to alternatives [47]. There have been cases of using equipment such as an MRI for digital modeling; however, this is ill-suited for creating a digital twin. One of the most useful attributes of digital twins is their ability to continuously update in real time. This requires the sensors to be capable of continuously recording and uploading data to ensure the twin stays updated. As a result, an MRI would be insufficient for creating a digital twin of a human body. Sensors in implants are another alternative that has been used to create digital twins. Implant sensors are sensors that can gather data by being placed within the human body. Being placed within the body is advantageous for measuring certain parameters, such as blood glucose levels, which can be used to treat diabetes. Also, by being operable inside the body, the implant can constantly record and upload data, allowing it to take advantage of the real-time capabilities of digital twins. For all their strengths, implants have downsides as well. Implants are inherently invasive, which may not be a feasible strategy depending on the individual. They are also typically more expensive than wearable devices and are more challenging to maintain. Wearable devices boast the ability to continuously record data, much like implants, while also being less invasive and easier to use. As a result, generating a digital twin using wearable sensors will provide a cost-effective and easily applicable method of data acquisition for an overall complicated process [48]. Smart wearable sensors are also a necessity for the digital twin to take advantage of the Internet of Things. With smart wearable sensors, it has become possible to constantly communicate across every device involved in the process of creating the digital twin [49].

In the context of healthcare, smart wearables are used to record biometric signals from the patient. These signals come from a variety of sources, depending on the needs of the patients as determined by the medical professionals. For example, wearable ECG devices are used to monitor heart conditions while EEG devices gather data related to neurological conditions [50]. Some sensors are used to monitor multiple parameters at a given time. Heart rate tracking devices are widely used as an indicator of general wellness to provide insight into the patient’s cardiovascular health [51]. Smart sensors contain a microcontroller unit, which is responsible for storing, analyzing, and transmitting data using a radio transmitter. The raw data gathered from the sensors must first be processed to derive meaningful information. This operation can be more rigorous depending on the type of sensor collecting the data. Regardless of the sensor being used, the initial step in processing always involves eliminating noise from the collected signal, which is accomplished by incorporating analog and digital filters. When utilizing EEG devices, it may be necessary to apply a filter that selects desired frequency bands. Once the data have been processed, they are transmitted to a software application on a paired device. 

To generate a digital twin, it is necessary to acquire data from the physical entity on which the twin is based. In many situations, the data used to construct a digital twin can be artificially generated using AI algorithms. This can be useful for constructing a digital twin for objects that may not exist yet and to test the behavior of machine learning algorithms and simulations [52]. For example, electrocardiogram results can be simulated to resemble data gathered from real patients. This can be further modified to emulate symptoms of heart conditions and develop algorithms that can detect said conditions, all without collecting data from any patients. Another additional use of generative AI is predictive modeling based on acquired sensor data. This can be useful for generating data on certain parts of the digital twin based on tangentially related input data from sensors [53]. There are different methods of using generative AI for the construction of human digital twins. Of interest is the generative adversarial network, which involves two competing neural networks that work together to create data samples that resemble genuine data [54]. The first network generates data samples based on an authentic distribution, while the second examines the generated data for discrepancies. Another way of generating data is by using a variational autoencoder. Like generative adversarial networks, variational autoencoders use two neural networks, the encoder and decoder, to generate artificial data to resemble a genuine data set. The variational autoencoder can accept input data and produce a dataset based on the input. This has been used in the context of constructing human digital twins by predicting body orientation and movement based on collected data from wearable sensors on just the hands and head [55]. 

Incorporating AI algorithms into the data acquisition and generation process can create a more robust digital twin for healthcare. It accomplishes this by complementing a weakness that exists when some datasets are too small when gathered from wearable sensors only. Some wearables can only be utilized for a limited amount of time, leading to an insufficient quantity of data to produce a digital twin at the beginning stage. Therefore, AI can be utilized to identify patterns, generate synthetic data, and make predictions based on sensor-gathered data to expand the dataset used by the digital twin. An application of this is using a Particle Swarm Optimization algorithm to identify brain tumors through digital twins. The images of brain scans are processed through the algorithm and a digital twin is constructed based on the patient to identify if a brain tumor is present [56]. Additionally, AI can be used to generate data for parameters that are not easily measured by wearable sensors in situations where wearables may be infeasible. For example, a digital twin has been developed for use in treating type-1 diabetes by monitoring glucose levels through the measurement of heart rate. This was accomplished using a KNN algorithm to train the digital twin to recognize correlations between glucose levels and heart rate patterns. This has allowed for a method of indirectly monitoring glucose concentrations through an easily worn fitness tracker watch [23].

### 3.2. Musculoskeletal System

The musculoskeletal system of the human body consists of the skeleton along with all attached muscles, tendons, and ligaments. The general purpose of the musculoskeletal system is to provide structure and movement for the body. As such, the skeleton can be represented by the position and orientation of the body, while the musculature can be modeled by measuring the electrical activity during contraction resulting in movement. Positional and orientation data for the human digital twin can be measured using inertial sensor units. Inertial sensors consist primarily of a combination of accelerometers and gyroscopes. Accelerometers measure linear acceleration along three axes by comparing the force of the acceleration detected by the device using gravity as a reference. The structure of an accelerometer is like a standard capacitor, with one of the plates being a movable object. As the object moves due to changes in acceleration, the distance between the object and the plate changes, as does the capacitance. This results in a variable capacitance which can be measured to determine the acceleration from the sensor. Gyroscopes are devices used to measure orientation by detecting the rate of change in rotation along three, six, or nine axes. They are built like accelerometers, with a primary difference being that gyroscopes measure angular velocity rather than linear acceleration. This is carried out by placing the moveable object on a coil that moves in response to rotation. Together, accelerometers and gyroscopes are used to measure an object’s position, orientation, and movement as an inertial sensing unit. 

Inertial sensing units are commonly used in devices to detect body movements and positions and generate digital models. Examples of this include using contact sensors to create a digital twin of an arm. The digital twin captured the arm’s movement using two contact sensors with inertial sensing units. One sensor was placed on the upper arm and the other on the forearm. These results were verified by placing the same sensors on a six-axis robotic arm, which moved in a predetermined trajectory. The digital twin’s movement was nearly identical to the path traveled by the robotic arm [57]. Similar results have been found utilizing a robot exoskeleton to measure the movement of the wearer’s arm [58]. An alternative to the inertial sensing unit is the textile-based sensors used to make smart clothes [59]. These sensors operate by measuring the change in conduction in the textile material. As the material folds or deforms, the conduction in that area changes, which is measured. When used in smart clothes, folds produced during movement or general changes in orientation will cause an increase in conduction while producing a decrease in areas that get stretched [44]. This has been used as a method to track movements during exercise for a group of athletes wearing smart clothes and to create a digital twin that records the athletes’ movements [51].

Muscle activity is typically measured using electromyography. Muscle fibers consist of specialized cells that contract in response to electrical signals. Motor neurons deliver signals from the nervous system, which causes the desired muscle fibers to contract. These contractions can be measured using electrodes, which detect potential differences across the muscles. Electrodes with wearable data acquisition systems, used for electromyography, are a convenient form of wearable sensor that is inexpensive and easily applied to the desired area. Electrodes are placed on the beginning and end of each muscle group to measure specific activity. This information can be used for the digital twin to predict the muscle activities and movement of the human body. This works in conjunction with the inertial sensing unit to accurately model and predict the subject’s position and movement [60,61]. Digital twins representing the musculoskeletal system can also be used to predict the future development of musculoskeletal system disorders [62]. This can be performed by utilizing previously gathered data in conjunction with predictive modeling to identify common risk factors the subject may be experiencing.

### 3.3. Circulatory System

The circulatory system transports and delivers oxygen throughout the human body via the blood. The heart is the central component of the system, as it controls the flow of blood based on the body’s needs. Some of the vital measurements that can be obtained include heart rate and blood oxygen levels, both of which can be measured through wearable sensors. The heart rate is an indicator of general health and a possible detector of abnormalities. Abnormalities in the frequency or rhythm of the heartbeat can suggest possibly fatal conditions that require immediate attention. Blood oxygen levels are crucial in maintaining good health. Maintaining a satisfactory blood oxygen level ensures the proper functioning of the organs throughout the body. When the blood oxygen level is too low, complications may ensue. These complications may include confusion and unconsciousness, resulting in the need for intervention, such as respiratory therapy or life-saving measures. Using wearable sensors can detect abnormalities in real time as problems occur, which can often happen with little notice.

There are a variety of commercially available wearable sensors to measure heart rate and blood oxygen levels. This is a common feature in almost every smart watch or fitness device. Two methods are typically used to measure heart rate: photoplethysmography and electrocardiography. Photoplethysmography utilizes an infrared sensor to measure the expansion of blood vessels through the skin. The expansion corresponds to a single heartbeat, which can be used to calculate the heart rate in beats per minute. Electrocardiography involves measuring the potential difference around the heart itself during contraction. This provides a more detailed measurement of heart activity compared to photoplethysmography but is more cumbersome due to the equipment needed, which can also be a cost factor. Blood oxygen levels can be obtained through pulse oximetry, which uses infrared lights to measure blood color. The measurement calculates how the blood in the vein interacts with the infrared light by recording how much of the light is reflected. Incorporating circulatory system data in the digital twin provides a method for constantly monitoring the subject’s vital signs [63]. Along with the data collected by the digital twin, hereditary information can also be added by user input to identify any early warning signs of said conditions, should they arise [64]. 

### 3.4. Nervous System

The nervous system is responsible for receiving and transmitting signals throughout the body through neurons; it is the primary method of the brain communicating with the rest of the body and receiving sensory information from the environment. Dysfunction of the nervous system can lead to epilepsy, stroke, or Parkinson’s disease, as well as other conditions [65]. Due to the complexity of the brain, imaging is typically carried out through a computed tomography (CT) scan or a magnetic resonance imaging (MRI) scan. Currently, wearable sensors are not sufficient to capture every faculty of the brain in great length, but some details may be measured this way [66]. 

Two common methods for measuring brain activity through wearable sensors are electro-encephalogram (EEG) and Functional Near-Infrared Spectroscopy (fNIRS) [67]. An EEG utilizes electrodes placed at numerous different locations on the scalp to measure brain activity. The results of an EEG are recorded as waves corresponding to each attached electrode. These electrodes measure voltage in different regions of the brain. If the waveforms are abnormal, it may suggest a healthcare concern, such as epilepsy. The fNIRS sensor is similar to the EEG, except it uses near-infrared (NIR) sensors (light sources and detectors) instead of electrodes to gather data. The NIR sensors measure the amount of hemoglobin, which carries oxygen throughout the body. Different concentrations of hemoglobin can be measured by the amounts of NIR light absorbed from the NIR source. Having too much or too little hemoglobin may also warrant the need for further evaluation to address health concerns. Utilizing brain wearables to generate digital twins can be used to detect strokes as they happen to expedite treatment [50]. The same data acquisition methods can be used to measure mental health parameters as well, along with user-inputted data [68,69]. With this, a mental health component can be added as a system as part of the human digital twin [70].

## 4. Model Generation

Once the data have been received from the sensors, the creation of the digital twin can begin. Data from the sensors are transmitted to a server where they are used to generate the digital twin [71]. Generation involves utilizing AI and machine learning algorithms to construct a model based on the parameters of the subject [72]. This also includes the act of processing the acquired data to interpret useful information. An example of this would be utilizing Deep Learning (DL) to analyze X-ray results to detect respiratory infections to include in the digital twin model [73]. In the context related to human digital twins, DL methods are useful for generating 3D models of body parts [74,75]. Of course, the model will continuously be updated based on new data as they are collected. Also, digital twins are based on sensor data and data manually inputted directly by the user. This is useful for information that is not typically collected from smart sensors or information that does not need to be updated continuously. An example of the former could include results from a questionnaire during a medical trial. A similar process has been used to model a patient’s mental state using their social media activity. Examples of the latter include information like medical history, genetic information, or family history of disease. Genetic information was included in digital twins to monitor cancer progression [76,77]. 

Data acquired from the smart sensors are uploaded wirelessly to a data storage service. From here, the data can be stored in different categories based on their origin and purpose. The data can be uploaded to a cloud server which will allow them to be accessed across various devices. As the model is being stored, the data service is still continuously receiving updates to have the stored data match the physical counterpart [78]. At this stage, the data from the sensors can be processed to generate a more complete digital twin [79]. Machine learning plays a crucial role in the process of generating a digital twin. Data collected from sensors are often not entirely sufficient to create a digital twin. Once the data from the sensors are received, machine learning can be used to make predictions about the system based on the limited data received [80,81]. One such case of this would be in a digital twin predicting locomotion. Typically, data are taken from sensors for a specific body part indicating movement. However, to generate a digital twin that represents the movement of the entire body, predictive modeling is used to generate data for the rest of the body. This has been shown to be successful in the case of modeling full-body exercises based on soft strain sensors [44]. Utilizing a convolutional neural network (CNN), the digital twin can be trained to recognize patterns in the acquired data to report significant findings. This has been used in digital twins when processing genetic information from the patient when generating a model. The CNN can then utilize this information when treatment recommendations are made by ruling out potentially hazardous options [82]. Creating a virtual avatar to visualize the model can be achieved through various modeling software such as Unity [83] or Make Human [84].

Human digital twins contain a great deal of sensitive information about individuals, so security is a necessary consideration as well. The significance of this concern is only increased due to the incorporation of digital twins into IoT healthcare systems. With more systems being interconnected, more opportunities arise for security and data integrity compromises [85]. Contents of the digital twin can be secured utilizing Blockchain to ensure privacy [86]. This has been utilized to secure the contents of not only patient sensor data but also correspondence with healthcare professionals. Another model involves employing federated learning to encrypt patient data to ensure privacy while the information is being transmitted to different medical facilities [87]. This method is also capable of fault detection, whether that be in the sensors that collect patient data or any possible loss of information during processing or transmission.

## 5. Discussion

The literature presented in this review supports the possibility of creating a human body digital twin using wearable sensors. Most of the current research is focused on creating digital twins of specific body parts or individual organs instead of the entire body. This can be attributed to a lack of simultaneous data and greater computational demands for generating a model for the entire body instead of a single, isolated area. Specifically, trying to create a digital twin that can accommodate every system in the human body would require an infeasible level of processing capability. By focusing on the musculoskeletal, circulatory, and nervous systems, an accurate, reasonably detailed digital twin can be created that represents a human body [Figure 4]. A human digital twin created in this manner could prove to be a versatile asset in the healthcare industry. 

**Figure 5:**
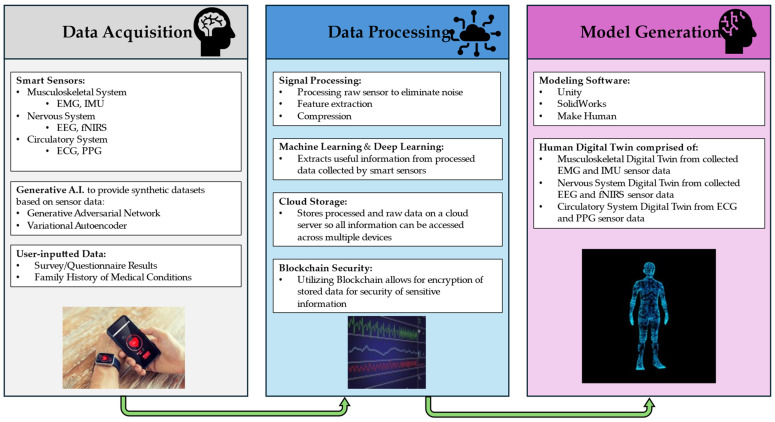
Block diagram representing the model generation process for a human digital twin.

A proposed method for generating a human digital twin can be found in Figure 5. This process is comprised of three primary steps: data acquisition, data processing, and model generation. The data acquisition section consists of the preliminary steps in gathering data to be used for the creation of the human digital twin. Smart sensors are used to capture information pertaining to the musculoskeletal, nervous, and circulatory systems. Additionally, generative AI can be used in conjunction with the physical sensors to add additional synthetic datasets that complement the acquired data. This process would also include any user-input data that could not be captured by sensors. Data processing involves transferring the acquired data into useful information that can be used for the model’s generation. The incoming data would need to be processed and filtered initially to eliminate noise and allow the data to be more compatible (feature extraction) with machine learning algorithms. Once the data have been processed, machine learning and deep learning algorithms are used to extract meaning from the acquired data, such as heart rate from ECG results or blood pressure from PPG results. It is also important to store this information on a cloud server such that it can be easily accessed across multiple devices, such as those belonging to the subject as well as the provider. Blockchain can also be integrated during this step to ensure the privacy of the subject due to the sensitive nature of the involved medical data. Finally, the processed data can be used to generate a model of the human digital twin. This model essentially will consist of three separate but related models of the musculoskeletal, nervous, and circulatory systems. This can be accomplished through a variety of modeling software such as Unity, SolidWorks, or Make Human. These models can utilize the aforementioned machine learning and deep learning algorithms that allow for a robust digital twin model that updates in real time with the subject and is capable of predictive modeling.

Table 2 presents a summary of the literature pertaining to the creation of human digital twins using wearable sensors. In each case, the objective of the research was met by creating a functional human digital twin, showing that a variety of wearable sensors can accurately model various systems present in the human body. Some articles also showcase the versatility of using data collected from one sensor to make accurate assumptions on multiple aspects of the human body. Each article focuses primarily on at least one type of wearable sensor to acquire the desired parameters to construct the digital twin. As a result, the function of the generated human digital twin is focused on serving a specific purpose such as tracking movement or measuring heart activity. Some of these studies show significant promise in healthcare applications. Of particular interest is the study by Elayan et al. that presented a digital twin to detect abnormal heart behavior based on wearable ECG sensors. This allows for the created digital twin to identify and classify abnormal heart rhythms in real time, allowing for faster treatment and improved treatment outcomes [49]. A study by Noei and Lakany involved utilizing a digital twin to control a wearable robotic arm to assist in rehabilitation. The wearable arm was outfitted with EMG sensors that trained a digital twin to detect muscle activation intention and assist in arm movements. This was used to assist in rehabilitation for patients with spinal injuries [58]. Similarly, Lauer-Schmaltz et al. presented a method of utilizing sEMG sensors to generate a digital twin that could assist in stroke rehabilitation. The created digital twin would record exercises from the patient to monitor rehabilitation progression. Notably, the digital twin was designed to be used by informal caregivers during assistance at the patient’s home. This showcases one of the most useful aspects of digital twins used in healthcare, in that the patient can receive a comparable level of care from medical professionals while in the convenience of their own home [50]. While these methods are suitable for the referenced digital twins, they do not offer a holistic view of the human body. No system in the human body acts in isolation; they all affect each other and work in concert. Thus, there is a need for further investigation in utilizing the different systems in the human body and gathering information on how they interact with each other to build a more complete picture. Such a method can be found as the proposed model in Figure 4. This method is differentiated from other commonly used methods of creating digital twins due to its varied and interconnected multi-modal data from which it is based. Human physiological systems are interconnected. By combining biometric data from multiple physiological processes, a more complete human DT model representative of the patient can be achieved. This allows the model to be more versatile in its applications and can be used as a multifaceted representation of the patient for any desired need. This would account for a problem of some digital twin healthcare applications where the digital twin is highly specific and not reusable. Instead, this new model would be a running health record of the patient that can be referred to by healthcare professionals as a reference when treating their patients.

Currently, frequently used wearables to generate digital twins in a healthcare environment include commercially available smart watch devices. Smart watches have proven to be an excellent choice for digital twin generation due to their relative affordability and ease of wear. Examples of these include the lines of Fitbit and Apple Watch products [88]. Since these are typically always worn, even when sleeping, there is an abundance of data always being collected, which provides a highly detailed model. They are also designed to work with a dedicated application on smart phones to store data and provide analytics for the user. These are capable of continuously recording heart rate information and uploading the data to a cloud server where it can be processed to determine vital information about the patient. Such vital information includes heart rate, arrhythmias, sleep patterns, and activity detection. 

Digital Twin technology is still very much in its infancy and is rapidly progressing with new advancements. One of the remaining limiting factors in a wearable-based approach to digital twins in healthcare, however, lies in the efficiency of the required wearables to obtain data from multiple human physiology systems in a cohesive way that is not physically uncomfortable for the patients themselves. Some devices currently are a bit cumbersome to wear and thus can only take data for a limited span of time. As such, the data gathered from the patient are limited as well. Naturally, designing a sensor that is more comfortable for the patient to wear and allowing them to wear them for a longer period, would provide a better digital dataset to generate a digital twin. One example of such a device would be one that utilizes an ultrasonic sensor to measure cardiovascular activity across the entire body rather than at one point [44]. Advancements in smart cloth technologies have also shown to be capable of acting as multiple sensors at once, effectively acting as an EMG and ECG sensor that can be worn more conveniently for the patient [59].

**Figure 6:**
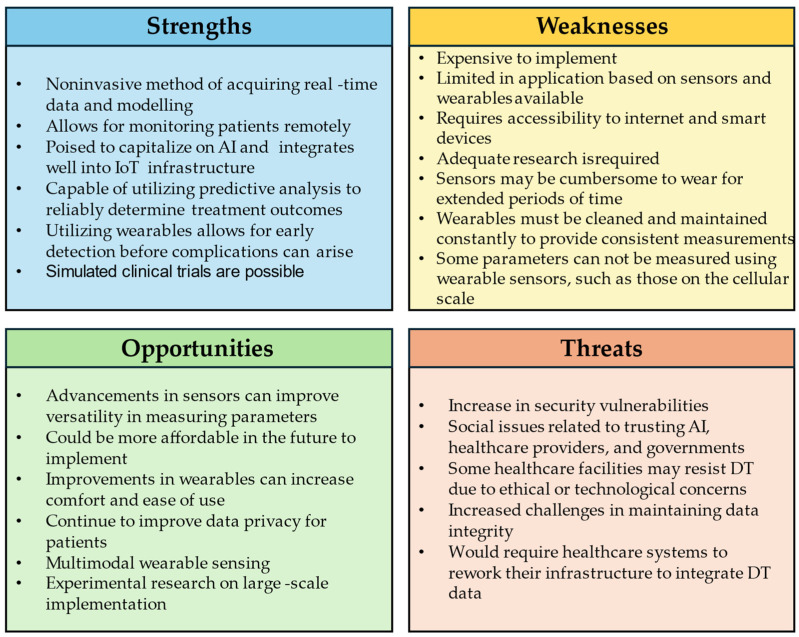
SWOT Analysis pertaining to the feasibility of utilizing digital twins for healthcare applications using wearables.

The greatest strength of digital twins is their ability to update and stay consistent with the data of their physical counterparts. As such, a human digital twin would be invaluable for establishing a baseline of general health, as well as detecting early warning signs in a timely fashion compared to waiting for physical symptoms to appear. Also, this would allow for remote monitoring of the patient’s condition, which would be useful to meet the patient’s convenience needs, in that they would not have to physically attend a face-to-face session with a medical practitioner [89]. With the advent of COVID-19, the need for personalized, remote medicine has caused a greater interest in the possibilities of human digital twins [90]. Another benefit lies in the cost-effectiveness of wearing sensors in lieu of a patient being admitted to a hospital for monitoring. This can greatly increase the versatility and effectiveness of point-of-care testing. With the aid of digital twins, point-of-care testing can provide useful data analytics to the patient’s healthcare team in real time that can be used to further assist the patient. The information from the digital twin can provide faster results for diagnoses or help determine if the patient’s current treatment plan is effective. This, in turn, allows the patient to have timely access to better healthcare at their convenience. This can be crucial in locations where healthcare access may be more challenging since it is entirely remote [91]. Similarly, digital twins allow for more opportunities for remote treatment such as rehabilitation, which may be more convenient for the patient if travel is difficult or infeasible. Figure 6 presents a SWOT analysis for utilizing digital twins with wearables in healthcare applications. Additionally, human digital twins are beneficial in other fields besides healthcare. Having a human digital twin of operators in industrial settings can provide useful information on safety concerns with how the operator interacts with the present machines [82,92,93,94,95]. This has also been used to simulate human–robot interactions in factories as well [96,97,98,99,100]. Human digital twins are also an area of interest due to increasing research into virtual and augmented reality [101], where digital twins can serve as virtual avatars [91,102].

## 6. Conclusions

This review paper presents possibilities for generating a holistic human body digital twin. Currently, the greatest challenge in generating detailed digital twins is computational power and the availability of sensors that can simultaneously collect data from humans. Digital twins are well suited to modeling specific objects or contained systems. However, the human body is a collection of dynamic systems that depend on each other. As a result, accurately modeling a human body requires a great deal of smart sensors and generous assistance from AI. In the case of wearable sensors, as more systems of the human body are being analyzed, more sensors are required. Most wearable sensors serve a specific function, so many different sensors would be required based on how detailed the desired model would be. This can become cumbersome for the subject to wear, especially for longer periods of time. However, if new wearables were developed that could capture a wider variety of data while remaining compact, then human digital twins could become more feasible. Further research is required to generate a digital twin of the human body. The human body is comprised of many dynamic systems, all of which interact with each other. Attempting to model each system independently may omit essential data for overall wellness. Furthermore, advances in wearable sensor technology could provide the acquisition of data that are reliable for generating a detailed human digital twin. Incorporating digital twins into healthcare has its own strengths, but challenges remain. Some noted barriers are insufficient research, the cost of equipment, wearable sensors and related technologies, access to the internet, and ensuring privacy and confidentiality. Depending on the patient’s circumstances, it may not always be feasible to collect data with wearables for extended periods of time. To take advantage of this technology, it is important to be able to accommodate patients with the necessary equipment they need to collect data whenever is convenient for them. Digital twins in healthcare can also lead to more vulnerabilities in the patient’s data. As such, security is of the utmost importance when utilizing digital twins. Utilizing wearables to create digital twins allows for a great deal of convenience in treating patients as care plans can be administered remotely. While this carries many benefits, it also opens the unfortunate possibility of fraudulent activity, a problem that is arising with the increasing shift towards digitalization. As a result, it is also important to always confirm the patient’s identity remotely and by having routine in-person check-ups in addition to maintaining the digital twin. Additionally, having a proper verification method for when the data are accessed by the patient or healthcare professional is necessary to ensure confidentiality.

Digital twins have a great deal of potential to revolutionize the healthcare industry. Not only can they provide a holistic view of patients, but they provide access to many who are located in rural areas, including those that are economically disadvantaged. Advancements in smart sensors will lead to better quality data from which to build the digital twin, leading to datasets that are more accurate. Similarly, advancements in AI and machine learning will result in competent and robust models that can enhance the patient’s healthcare. 

## Figures and Tables

**Table 1:** Table including the databases, key terms, and years of publication for the literature reviewed.

Field	Content
Database	Google Scholar, IEEE Xplore Digital Library, Materials Science & Engineering Collection, ScienceDirect, ACM Digital Library, PubMed
Key Terms	“Digital Twin”, “Human Digital Twin”, “Wearable Sensors”, “Digital Modelling”, “Human Digital Modelling”, “Digital Twin Healthcare”
Year of Publication	2018–2024

**Table 2:** Summary of relevant work on human digital twin generation using wearable sensors.

Ref	Objective	Wearable Used	Data Collected by Wearable	HDT Function
[8]	To utilize human digital twins to improve safety for workers in manufacturing systems	Inertial (MOCAP System)	Movement and position data from workers	Determine based on inertial data if a disturbance occurred in the workspace
[33]	To develop an affordable and user-friendly wearable system to produce human digital twins	Inertial (9-axis motion tracking system)	Tracks movement and position	Generates a human digital twin capable of tracking the subject’s movements and produces a 3D virtual model
[49]	To use ECG data to detect and predict heart conditions as they arise	ECG (through smart watches)	Heart rate to detect abnormalities such as arrhythmia	A digital twin was created based on ECG data that could identify and diagnose heart problems in real time for the patient
[57]	To generate a human twin that can be used to detect certain poses of the subject	Inertial (9-axis motion tracking system)	Measures orientation to detect certain poses	Generates a 3D model of a human arm based on movement data gathered from an IMU system on the subject’s arm
[58]	To utilize a wearable robotic exoskeleton to assist patients with arm movements during rehabilitation	EMG sensors within the robotic exoskeleton	Utilizes EMG sensors to measure muscle activity intent	Assists movements of the patient’s arm using a digital twin created from EMG data to detect muscle activation intent
[59]	To develop a smart clothing system that utilizes a variety of smart sensors to produce a digital twin of the wearer	MAX30102, MAX90614, WTGAHRS2, ATK1218-BD	Measures heart rate, blood oxygen levels, body temperature, movement, and position	Generates a human digital twin based on the wearer’s data collected from the wearable sensors and provides audio feedback and changes the temperature of the clothing
[44]	Review of novel wearables that have been used to generate digital twins	Various experimental IMU and EMG sensors	Measures movement and muscle activation	Digital twins created were able to measure the locomotion and position of the wearer based on movement from one part of the body
[51]	Utilizes human digital twins to analyze the fitness parameters of athletes to evaluate and predict performance	Fitbit Charge HR (heart rate sensor)	Measures heart rate data to record exercises and sleep activity	Human digital twins were created based on the athlete’s fitness data gathered from their Fitbit and inputted data through MyFitnessPal to predict exercise outcomes and offer recommendations on improving performance
[50]	To develop a user-friendly dashboard that can be used by informal caregivers to monitor the progress of stroke rehabilitation	sEMG (surface electromyography)	Measures muscle activation intent in the upper limb	Human digital twins were created based on sEMG that could monitor muscle activity in the upper limb during stroke rehabilitation
[68]	To develop a digital twin that represents a subject’s stress level primarily based on wearable sensors, phone usage, and social media activity	Smart watch (heart rate sensor and exercise tracker)	Measures heart rate data to form a correlation with phone and social media data to detect anxiety levels	The generated human digital twin could identify mental health conditions as they develop in response to stressors caused by COVID-19

## Data Availability

No new data were created or analyzed in this study. Data sharing is not applicable to this article.
